# Quality of Life in Short Stature: Comparisons Between Normal Variants Short-Statured and Normal-Statured Children and Adolescents and Agreement with Their Parents

**DOI:** 10.3390/healthcare13172213

**Published:** 2025-09-04

**Authors:** Anna Guerrini Usubini, Nicoletta Marazzi, Laura Abbruzzese, Adele Bondesan, Graziano Grugni, Gianluca Castelnuovo, Alessandro Sartorio

**Affiliations:** 1 Experimental Laboratory for Auxo-Endocrinological Research, Istituto Auxologico Italiano, IRCCS (Istituto di Ricovero e Cura a Carattere Scientifico), 28824 Piancavallo-Verbania, Italy; n.marazzi@auxologico.it (N.M.); a.bondesan@auxologico.it (A.B.); sartorio@auxologico.it (A.S.); 2 Division of Auxology, Istituto Auxologico Italiano, IRCCS (Istituto di Ricovero e Cura a Carattere Scientifico), 28824 Piancavallo-Verbania, Italy; l.abbruzzese@auxologico.it (L.A.); g.grugni@auxologico.it (G.G.); 3 Psychology Research Laboratory, Istituto Auxologico Italiano, IRCCS (Istituto di Ricovero e Cura a Carattere Scientifico), 28824 Piancavallo-Verbania, Italy; gianluca.castelnuovo@auxologico.it; 4 Department of Psychology, Catholic University of Milan, 20123 Milan, Italy

**Keywords:** short stature, quality of life, QoLISSY, child/adolescent report, parent report, constitutional growth delay, familial short stature

## Abstract

Background/Objectives: This study aimed to evaluate quality of life in children and adolescents with normal variants of short stature compared to age- and sex-matched individuals with normal stature and to assess the agreement between children/adolescents-reported and parent-reported outcomes. Methods: A total of 65 child–parent dyads were enrolled, including 29 children and adolescents with short stature (15 males, 14 females; mean age: 11.2 + 2.0 years; mean height standard deviation score, HSDS: −2.10 + 0.57) and 36 children and adolescents with normal stature (19 males, 17 females; mean age: 11.3 + 1.93 years; mean HSDS: 0.56 + 0.78). Quality of life was assessed using the Quality of Life in Short Stature Youth (QoLISSY) questionnaire. Statistical analyses included independent samples *t*-tests, and effect sizes were computed using Cohen’s *d*. Results: Among short-statured children and adolescents, no significant correlations were found between HSDS and all domains of quality of life. Short-statured children and adolescents exhibited significantly lower QoL across all domains compared to their normal-statured peers. Coping was higher in children and adolescents with short stature compared to their peers of normal stature. Similarly, parents of short-statured children and adolescents perceived a lower QoL for their sons and daughters and reported greater concern about the future and a more perceived personal impact than parents of normal-statured children and adolescents. No statistically significant differences were found between sons/daughters and parent reports, indicating a relatively high level of agreement in quality of life (QoL) perceptions. Conclusions: These findings underscore the psychosocial impact of short stature and highlight the importance of incorporating both child and parent perspectives in the clinical assessment.

## 1. Introduction

Short stature in children and adolescents [1] can result from various conditions, including the most frequent forms of familial short stature (FSS) and constitutional growth delay (CGD), also known as normal variants of short stature, growth hormone deficiency (GHD) [2], idiopathic short stature (ISS) [3], Turner Syndrome [4], being born small for gestational age (SGA) [5], and SHOX haploinsufficiency [6]. While the physical characteristics of the normal variants of short stature are well documented [7], their psychological and social implications remain subjects of ongoing debate and investigation.

Several studies have reported that children with short stature may face psychosocial challenges [8,9], including stigmatisation, bullying [10], social isolation, low self-esteem, behavioural problems, social immaturity, infantilization, and lower academic achievement. These difficulties may contribute to internalising behaviours, such as depressive symptoms [11]. However, other studies suggest that not all children with short stature experience significant psychological distress [12,13], highlighting inconsistencies and controversies within the literature.

Further complexity arises from discrepancies between child-reported and parent-reported quality of life. A systematic review by Backeljauw and colleagues [14] noted that while some studies report strong agreement between child and parent assessments [15,16], others showed low concordance between the two perspectives [17].

Understanding the parental perspective on the impact of short stature on their child’s quality of life (QoL) is crucial. It can help identify unmet needs, improve communication between clinicians and families, capture the broader societal impact of the condition, and enable more personalised and effective care [18]. Nevertheless, studies focusing specifically on the agreement between parent and child assessments of quality of life (QoL) in the context of short stature remain limited, both in terms of number and scope. Additionally, most existing research has been conducted outside of Italy, which limits the applicability of the findings to different cultural and healthcare systems.

Given these gaps and ongoing debates, the present study aimed to compare QoL assessments made by children and adolescents with normal variants of short stature and those with normal height and their parents, respectively, using the Quality of Life in Short Stature Youth (QoLISSY) questionnaire. 

## 2. Materials and Methods

### 2.1. Participants and Procedures

The sample was composed of 29 dyads of participants (15 males, 14 females, mean age ± SD: 11.2 ± 2.0 years; mean height Standard Deviation Score, SDS: −2.10 ± 0.57) with normal variants of short stature (height < 10th centile for age and sex, using the Italian reference growth charts) [19] and one of their parents, and 36 dyads of normal-statured age-and sex-matched individuals (19 males, 17 females, mean age ± SD: 11.3 ± 1.9 years; mean height SDS: 0.56 ± 0.78) and one of their parents.

Within the subgroup of normal variants of short stature, 20 participants had familial short stature (FSS), and 9 had constitutional growth delay (CGD). FSS was characterised by short stature observed in other family members (not necessarily the parents), height within the expected range for the parental target height, proportionate physical appearance without significant clinical signs, normal timing of pubertal development, and bone age corresponding to the chronological age. CGD was characterised by a negative familiarity for short stature (i.e., “short for their parents”), a proportionate physical appearance without significant clinical signs, absence of any systemic, endocrine, nutritional, or chromosomal abnormalities, and delayed bone age. A normal GH responsiveness during at least one GH-releasing stimulus (i.e., GH peak > 8 ng/mL) excluded the presence of GH deficiency (GHD) in 10 short-statured participants with SDS lower than—2.0 who were clinically considered worthy of this investigation. Inclusion criteria for the two subgroups were: (1) being Italian and Caucasian; (2) being aged between 12 and 17 years; (3) providing informed consent to participate. Exclusion criteria included any physical or psychological impairment that could compromise participation in the study. Participants with the normal variants of short stature and their parents were consecutively recruited at the Research Center for Growth Disorders, Istituto Auxologico Italiano, IRCCS, Milan, Italy, between April and June 2023. Participants with normal stature and their parents were recruited in the same period from the sons and daughters of the hospital’s medical, research, and administrative staff, as well as friends. Once enrolled in the study, participants and their parents were asked to complete a child- and parent-reported questionnaire on quality of life, as described in the subsequent section. The study was approved by the Ethical Committee of Istituto Auxologico Italiano, IRCCS, Milan, Italy (approval number EC: 2023_03_21_03; date of approval: 21 March 2023; research code: 01C312; acronym: PSICOSHORT). The research was conducted in accordance with the Declaration of Helsinki and its subsequent revisions.

### 2.2. Measures

Demographic data (i.e., gender, age) were self-reported. Weight and height were measured by the internal medical staff. Standing height was determined by a Harpenden Stadiometer (Holtain Limited, Crymych, Dyfed, UK). Weight was measured to the nearest 0.1 kg using an electronic scale (RoWU 150, Wunder Sa.bi., Trezzo sull’Adda, Italy). Body mass index (BMI) was calculated using the formula kg/m^2^.

To assess the quality of life in individuals with short stature, the core modules of the Quality of Life in Short Stature Youth (QoLISSY) questionnaire [20] were used. For all licensing and permission requests for QoLISSY, please contact IQVIA at IQVIA_COAs@iqvia.com. The use of the Licensed Materials in the form provided by IQVIA has been allowed only in connection with the current project (“PSICOSHORT”) developed by Istituto Auxologico Italiano. The questionnaire was administered only by the principal investigator (A.S.) or his coworkers to patients/controls participating in the project.

The QoLISSY is a condition-specific, self-report instrument designed to capture the psychosocial impact of short stature from the perspectives of children and adolescents aged 8–18 years and their parents [21]. It includes both child self-report and parent versions, ensuring a multidimensional evaluation of quality of life. The child version consists of 50 items rated on a 5-point Likert scale, assessing three core domains of quality of life: Physical, Social, and Emotional quality of life, as well as three additional domains: Coping, Beliefs, and Treatment. The sum of the mean scores of the three core domains provides a general measure of quality of life (total score). Higher scores indicate a better perceived quality of life. For this study, the “Treatment” subscales were not administered as our participants were not under any medical treatment. The parent version is used to obtain parent reports on identical domains and assesses two additional parent-specific domains: “Future” (items about the child’s future) and “Effects on Parents” (11 items about the effects of their child’s short stature on parents). The response scale consists of a five-point Likert scale ranging from “not at all/never” to “extremely/always”. Psychometric properties of the original QoLISSY revealed good-to-excellent internal consistency, with Cronbach’s α ranging from α = 0.82 (coping scale) to α = 0.92 (total score) in the self-report and from α = 0.86 (Physical scale) to α = 0.95 (Total score) in the parent report. For this study, the Italian-validated version was administered. Scoring was performed according to the standard manualised procedure, with domain and total scores transformed to a 0–100 scale. In our sample, the internal consistency of the scales (α) was good, ranging from 0.594 (“Social scale”) to 0.901 (“Emotional scale”).

### 2.3. Statistical Analysis

The sample size estimation was performed using GPower (version 3.1.9.7). Based on a two-tailed independent samples *t*-test with α = 0.05, power = 0.80, and a large effect size (*d* = 0.80), the required sample size to correctly detect a statistically significant difference between groups was determined to be 26 participants per group (total sample = 52).

Continuous variables were presented as means and standard deviations, while categorical variables were presented as frequencies and percentages. The normality of the distribution of the variables has been assessed using the Skeweness and Kurtosis indices. To assess quality of life, comparisons of QoLISSY child and parent versions in both short-statured and normal-statured individuals were performed using independent sample *t*-tests (in case of unequal variances, Welch’s corrected test was used). Additional Pearson’s r correlations (or proper non-parametric variant) were computed to address the associations between child and parent reports of QoLISSY in both short-statured and normal-statured individuals. Critical alpha was set at 0.05. Cohen’s *d* was used as a measure of effect size with the following interpretation: *d* = 0.2: small effect; *d* = 0.5: medium effect; *d* = 0.8: large effect [22]. Analyses were performed using Jamovi (version 2.6.26).

## 3. Results

The sample was composed of 29 short statured individuals with normal variants of short stature (i.e., FSS and CGD, 15 males, 14 females, mean age ± SD: 11.2 ± 1.96 years; mean height SDS: −2.10 ± 0.57) and their parents (*n* = 29) and 36 age- and sex-matched normal-statured children (19 males, 17 females, mean age ± SD: 11.3 ± 1.9 years; mean height SDS: 0.56 ± 0.78) and their parents (*n* = 36). More participants than the minimum required were enrolled in order to account for potential dropouts due to incomplete questionnaires. In the short-statured subgroup, 68% of the dyads came from Northern Italy, and 82% had a middle socio-economic status.

In the age- and-sex-matched normal-statured subgroup, all the dyads (100%) came from Northern Italy, and 69% had a middle socio-economic status.

All variables were normally distributed. Therefore, we proceeded with parametric statistics.

Since no significant differences were found in any of the subscales of the QoLISSY among short-statured individuals with FSS and CGD, the two populations were considered as a single subgroup. No correlations were found between the physical, social, emotional, and total scores of QoLISSY and HSDS in the short-statured subgroup. Descriptive statistics of the sample are reported in Table 1.

### 3.1. Comparisons Between Short-Statured and Normal-Statured Individuals by Using QoLISSY

Table 1 also shows the comparison between short-statured and normal-statured children and adolescents. Results indicated that normal-statured individuals reported significantly higher Physical QoL compared to short-statured individuals [*t*(63) = 4.74, *p* < 0.001], with a large effect size (*d* = 1.214).

Similarly, Social QoL scores were significantly higher among normal-statured individuals than their short-statured peers [*t*(63) = 6.63, *p* < 0.001], with a very large effect size (*d* = 1.730).

For Emotional QoL, normal-statured children also scored significantly higher than short-statured individuals [*t*(63) = 2.69, *p* = 0.009], indicating a medium effect size (*d* = 0.671).

Regarding the Coping subscale of QoLISSY, results showed higher scores in individuals with short stature compared to their normal-statured peers [*t*(63) = −2.06, *p* = 0.043], indicating a medium effect size (*d* = −0.515).

No significant differences were found between groups for the “Beliefs” subscale of the QoLISSY.

Finally, the total QoL score was significantly greater in the normal-statured group than in the short-statured group [*t*(63) = 5.44, *p* < 0.001], with a large effect size (*d* = 1.390).

### 3.2. Comparisons Between Parents of Short-Statured and Parents of Normal-Statured Children and Adolescents in QoLISSY

Independent samples *t*-tests were conducted to examine differences between normal-statured and short-statured individuals based on parent-reported QoL outcomes using the QoLISSY instrument. Results demonstrated that Physical QoL was significantly higher in the normal-statured group compared to the short-statured group [*t*(63) = 6.60, *p* < 0.001], with a very large effect size (*d* = 1.72). Similarly, social QoL was significantly greater among parents of normal-statured individuals than those of short-statured individuals [*t*(63) = 8.49, *p* < 0.001], reflecting a very large effect size (*d* = 2.22). In terms of emotional QoL, parents of normal-statured individuals reported significantly higher scores than parents of short-statured individuals [*t*(63) = 5.94, *p* < 0.001], with a large effect size (*d* = 1.53).

As far as Coping is concerned, results showed that parents of children and adolescents with short stature had higher scores than those of normal-statured subjects [*t*(63) = −2.69, *p* < 0.009], reflecting a medium effect size (*d* = −0.67). Similarly, in the Beliefs subscale, parents of individuals with short stature scored higher than those of individuals with normal stature [*t*(63) = 2.15, *p* < 0.036], with a large effect size (*d* = 0.53).

For the total QoL score, normal-statured individuals were rated significantly higher than short-statured individuals [*t*(63) = 7.85, *p* < 0.001], with a very large effect size (*d* = 2.04). Moreover, expectations regarding the future were significantly more optimistic in the parents of normal-statured subjects (M = 97.8, SD = 6.03) compared to those of short-statured individuals [*t*(63) = 5.06, *p* < 0.001], with a large effect size (*d* = 1.32). Finally, the effects on the parents’ domain revealed that the perceived impact on parents was significantly lower (i.e., more favourable) in the normal-statured group than in the short-statured group [*t*(63) = 4.96, *p* < 0.001], also reflecting a large effect size (*d* = 1.27). Results are presented in Table 2.

### 3.3. Comparison Between Individuals’ and Parents’ Reports in QoLISSY

Comparisons were also conducted to assess discrepancies between child–adolescent and parent-reported QoL scores for both normal-statured and short-statured subgroups across multiple domains of the QoLISSY instrument.

In the normal-statured group, no statistically significant differences were found between individuals’ and parents’ reports across any domain. Differences were small and non-significant for Physical QoL [*t*(35) = −1.858, *p* = 0.067, *d* = −0.438], Social QoL [*t*(35) = −0.965, *p* = 0.338, *d* = −0.228], Emotional QoL [*t*(35) = −1.032, *p* = 0.303, *d* = −0.243], and Total QoL [*t*(35) = −0.526, *p* = 0.601, *d* = −0.124]. Although the Physical QoL difference approached significance, the effect size remained moderate. Coping, Beliefs, and Total QOL scores revealed minimal variation, with all effect sizes close to zero. Similarly, in the short-statured group, child–parent differences were not statistically significant in any domain. For Physical QoL, the difference was negligible [*t*(28) = −0.169, *p* = 0.867, *d* = −0.044]. Modest, non-significant discrepancies were observed for Social QoL [*t*(28) = 1.385, *p* = 0.172, *d* = 0.363], Emotional QoL [*t*(28) = 1.655, *p* = 0.103, *d* = 0.434], and total QoL [*t*(28) = 1.141, *p* = 0.259, *d* = 0.299], with small to moderate effect sizes. Similarly, a modest effect was also detected in the Beliefs subscale [*t*(28) = 1.664, *p* = 0.102, *d* = 0.437]. Results are reported in Table 3.

Additionally, several Pearson’s *r* correlations were computed to address the associations between child and parent reports of QoLISSY in both short-statured and normal-statured individuals. Results confirmed significant correlations between individuals’ and parents’ reports across any domain except for the emotional domain, in which the correlation was not statistically significant. Results are presented in Table 4.

## 4. Discussion

The study compared quality of life (QoL) outcomes between short-statured individuals and their normally statured peers, as well as between their respective parents. It also compared individuals’ reports with those of their parents about quality of life (QoL) related to stature.

Although short stature is classically defined as a height that is two standard deviations (SDS) or more below the mean height for individuals of the same sex and chronological age in a given population, in the current study, we preferred to include also individuals with a lower degree of statural defect (i.e., <10th centile), in order to observe possible differences in Qolissy related to the different degrees of statural defect.

Since no significant differences were detected in all subscales of the QoLISSY between short-statured individuals with FSS and CGD, we were able to consider the two sub-populations as a single group. Similarly, no correlations were found between the physical, social, emotional, and total scores of QoLISSY and HSDS in the short-statured subgroups. These findings suggested that it was not the severity of short stature itself that impacted QoL, but rather the fact of being perceived as different from average-height peers. This result is notable, especially since patients with CGD usually also experience delayed pubertal development, which is assumed to exacerbate psychosocial burden [23].

Comparisons between short-statured and normal-statured individuals revealed that subjects with normal variants short stature have significantly lower QoL across all domains measured by the QoLISSY questionnaire compared to their normal-statured peers. These differences were statistically significant and clinically meaningful, as reflected in very large effect sizes, particularly in the social and physical domains. These findings are consistent with the existing literature. Bullinger et al. [20] consistently reported that having a height substantially lower than the norm for gender and age can be perceived as a social stigma that may potentially impact both self-perception and social integration. Additional studies have repeatedly shown that individuals with short stature often experience reduced well-being, particularly in social and physical functioning [17].

However, the results also showed that individuals with normal variants of short stature had significantly higher scores on the Coping subscale compared to their normal-statured peers, with a medium effect size. This finding suggested that, contrary to common assumptions, living with short stature could foster the development of adaptive coping strategies over time. These individuals may have developed a more active approach to managing challenges associated with their condition, relying on resilience, social support, and problem-solving skills [24]. A similar pattern has been documented among children with chronic illnesses when compared with healthy peers, who may nonetheless report comparable coping levels [25].

Parents of short-statured individuals reported lower scores in physical, social, emotional, and total QoL, with effect sizes ranging from large to very large, particularly in the social and total QoL domains. These findings closely align with previous findings addressing the perspectives of parents regarding the emotional and behavioural adjustment of their sons/daughters with short stature. Quitmann et al. [15] found that parents often perceived their children as experiencing more internalising problems, such as withdrawal and anxiety, compared to children of average height. These perceptions were particularly pronounced in individuals with short stature, as opposed to those who had achieved normal height. Additionally, parents reported lower QoL for their children, especially in domains related to social and emotional well-being.

However, in the Coping domain of the parent-reported QoLISSY, parents of individuals with normal variants of short stature reported significantly higher scores with a medium-to-large effect size. This finding suggests that parents perceive their short-statured children as having developed more effective coping mechanisms in dealing with their condition. Such awareness might hypothetically influence a better psychological adjustment of short-statured individuals. Further specific studies are required to explore this relevant hypothesis.

Conversely, in the Beliefs domain, parents of individuals of normal stature reported significantly more positive beliefs than parents of individuals with normal variants of short stature, with a moderate effect size. This may indicate that parents of short-statured individuals are more likely to hold concerns or uncertainties about the implications of their child’s condition, potentially reflecting worries about the condition of their sons/daughters.

In our study, the comparisons between child and parent reports of QoLISSY indicated no statistically significant differences between child–adolescent- and parent-reported QoL scores in either the normal-statured or short-statured groups across all QoLISSY domains. While some effect sizes showed modest trends, particularly in the short-statured group, these discrepancies did not reach statistical significance and generally fell within the small-to-moderate range. These findings suggest a relatively high level of agreement between parents and children–adolescents in their assessments of the child’s quality of life. To confirm this trend, correlations between child and parent reports on the QoLISSY showed positive and statistically significant associations across all domains, except for the correlation between emotional QoL reports from normal-statured children and their parents, which was not significant. All the observed correlations were modest. These results contrast with studies reporting significant parent–child incongruences in chronic health conditions. For example, in the study by Otero et al. [18], parents rated their children as having poorer physical and psychosocial QoL than the children themselves rated. However, some research has reported strong levels of agreement between children and their parents regarding the QoL they reported. According to Quitmann [15], the absence of significant discrepancies between child and parent reports may reflect a generally high quality of parent–child relationship and better child social support. The lack of correlation between child and parent reports in the emotional domain of QoLISSY can likely reflect a possible inconsistency in agreement according to the methods of analysis [26].

The current study presented some strengths and limitations. To the best of the authors’ knowledge, this is one of the first attempts to explore QoL in short stature by comparing short-statured and normal-statured children and adolescents’ reports on QoLISSY. In addition, by employing both the child–adolescent and parent versions of the QoLISSY questionnaire, the study provides a comprehensive and multi-informant perspective on QoL, capturing concordance between self-reports and parental observations or possible discrepancies in perception that can affect treatment adherence and psychological support. This dual-informant approach underscores the importance of integrating both patient and family perspectives in the assessment process, thereby enhancing shared decision-making and improving the overall quality of care. In addition, the results suggest that psychosocial interventions should be tailored not only to the individual needs of children and adolescents with short stature but also to the concerns and expectations of their families. Ultimately, these insights may inform the design of family-centred care strategies and multidisciplinary programs aimed at fostering resilience, enhancing psychosocial well-being, and ultimately improving the quality of life for individuals with short stature.

Furthermore, the study was conducted in the Italian context, where research on the psychosocial impact of short stature remains limited to date. Moreover, the use of the QoLISSY questionnaire, which is tailored specifically to individuals with short stature, may have enhanced the accuracy and comparability of both child and parent responses. Limitations concern the sample size, which may limit the generalizability of the findings, as participants were recruited from a single clinical setting. Secondly, the cross-sectional design prevents any causal inferences regarding the relationship between stature and QoL outcomes. Third, although the use of both the child and parent versions of the QoLISSY provides valuable insight, informants’ reports could reflect subjective biases, such as parental anxiety or social desirability, rather than objective differences in the child’s well-being. Additionally, the study may not account for relevant confounding variables, such as comorbid psychological conditions.

Future research should aim to expand the sample size and include participants from several settings to enhance the generalizability of the findings. Longitudinal studies are necessary to assess how quality of life changes over time in individuals with short stature (i.e., normal variants of short stature, such as FSS and CGD) and to determine whether specific interventions can enhance psychosocial outcomes. In addition, future studies should explore the role of moderating variables, such as family functioning, school environment, or peer relationships, that may influence the child’s adaptation to short stature. Integrating qualitative methods, such as interviews or focus groups, can also provide deeper insight into the lived experiences of both children and adolescents, as well as their families. Finally, cross-cultural comparisons using the QoLISSY across different countries could help to explore the psychosocial adjustment related to the normal variants of short stature (FSS and CGD) more in-depth.

## 5. Conclusions

Overall, our results underscore the psychosocial impact of short stature and highlight the importance of incorporating both child and parent perspectives in the clinical assessment of short-statured individuals in order to monitor (and/or improve, when necessary) their quality of life during their growth period.

## Figures and Tables

**Table 1:** Descriptive statistics of the sample and comparisons between normal-statured and short-statured children/adolescents in QoLISSY.

	Group	n	Means	SD	*t*	*p*	*d*
PhysicalQoL (C)	Normal-statured	36	94.4	9.61	4.74	<0.001	1.214
	Short-statured	29	77.6	17.1			
Social QoL (C)	Normal-statured	36	96.9	5.23	6.63	<0.001	1.730
	Short-statured	29	72.4	19.3			
Emotional QoL (C)	Normal-statured	36	94.9	27.58	2.69	0.009	0.671
	Short-statured	29	78.4	19.9			
Coping (C)	Normal-statured	36	36.9	25.08	−2.06	0.043	−0.515
	Short-statured	29	49.0	26.09			
Beliefs (C)	Normal-statured	36	73.4	19.73	−0.047	0.962	−0.011
	Short-statured	29	73.7	26.0			
QoLISSY Total (C)	Normal-statured	36	95.4	10.26	5.44	<0.001	1.390
	Short-statured	29	76.1	16.7			

Note: QOL: quality of life.

**Table 2:** Comparisons between parents of normal-statured and short-statured individuals in QoLISSY.

	Group	n	Means	SD	*t*	*p*	*d*
Physical QoL (P)	Normal-statured	36	97.8	5.03	6.60	<0.001	1.72
	Short-statured	29	78.3	15.2			
Social QoL (P)	Normal-statured	36	98.0	4.67	8.49	<0.001	2.22
	Short-statured	29	65.2	20.04			
Emotional QoL (P)	Normal-statured	36	93.4	10.13	5.94	<0.001	1.53
	Short-statured	29	69.9	19.3			
Coping (P)	Normal-statured	36	35.6	28.97	−2.69	0.009	−0.67
	Short-statured	29	53.0	21.08			
Beliefs (P)	Normal-statured	36	75.5	23.45	2.15	0.036	0.536
	Short-statured	29	62.5	25.33			
QoLISSY Total (P)	Normal-statured	36	96.4	5.07	7.85	<0.001	2.04
	Short-statured	29	71.1	16.7			
Future (P)	Normal-statured	36	97.8	6.03	5.06	<0.001	1.32
	Short-statured	29	78.3	20.01			
Effects on Parents (P)	Normal-statured	36	82.9	16.75	4.96	<0.001	1.27
	Short-statured	29	54.8	26.5			

Note: QOL: quality of life.

**Table 3:** Comparison between individuals’ vs. parents’ reports in QoLISSY.

	Group	n	*t*	*p*	*d*
Physical QoL (C-P)	Normal-statured	36-36	−1.858	0.067	−0.438
	Short-statured	29-29	−0.169	0.867	−0.044
Social QoL (C-P)	Normal-statured	36-36	−0.965	0.338	−0.228
	Short-statured	29-29	1.385	0.172	0.363
Emotional QoL (C-P)	Normal-statured	36-36	−1.032	0.303	−0.243
	Short-statured	29-29	1.655	0.103	0.434
Coping (C-P)	Normal-statured	36-36	0.203	0.840	0.048
	Short-statured	29-29	−0.735	0.465	−0.193
Beliefs (C-P)	Normal-statured	36-36	−0.408	0.685	−0.096
	Short-statured	29-29	1.664	0.102	0.437
QoLISSY Total (C-P)	Normal-statured	36-36	−0.526	0.601	−0.124
	Short-statured	29-29	1.141	0.259	0.299

Note: QOL: quality of life.

**Table 4:** Correlations between child and parent reports of QoLISSY in both short-statured and normal-statured individuals.

	Group	n	Pearson’s *r* Correlation Coefficient	*p*
Physical QoL (C-P)	Normal-statured	36-36	0.377	0.024
	Short-statured	29-29	0.356	0.058
Social QoL (C-P)	Normal-statured	36-36	0.514	0.001
	Short-statured	29-29	0.474	0.009
Emotional QoL (C-P)	Normal-statured	36-36	0.314	0.062
	Short-statured	29-29	0.361	0.054
Coping (C-P)	Normal-statured	36-36	0.411	0.014
	Short-statured	29-29	0.605	<0.001
Beliefs (C-P)	Normal-statured	36-36	0.619	<0.001
	Short-statured	29-29	0.465	0.011
QoLISSY Total (C-P)	Normal-statured	36-36	0.383	0.021
	Short-statured	29-29	0.422	0.027

## Data Availability

Raw data will be uploaded to www.Zenodo.org immediately after the manuscript’s acceptance, and they will be available upon reasonable request to the authors, A.G.U. and A.S.
